# Trace Elements in Alzheimer’s Disease and Dementia: The Current State of Knowledge

**DOI:** 10.3390/jcm13082381

**Published:** 2024-04-19

**Authors:** Magdalena Tyczyńska, Marta Gędek, Adam Brachet, Wojciech Stręk, Jolanta Flieger, Grzegorz Teresiński, Jacek Baj

**Affiliations:** 1 Department of Correct, Clinical and Imaging Anatomy, Medical University of Lublin, ul. Jaczewskiego 4, 20-090 Lublin, Poland; m.tyczynska@onet.pl (M.T.); wojciech.strek@gmail.com (W.S.); 2 Department of Forensic Medicine, Medical University of Lublin, ul. Jaczewskiego 8b, 20-090 Lublin, Poland; gedekmarta@gmail.com (M.G.); adambrachet@gmail.com (A.B.); grzegorzteresinski@umlub.pl (G.T.); 3 Department of Analytical Chemistry, Medical University of Lublin, Chodźki 4A, 20-093 Lublin, Poland; jolanta.flieger@umlub.pl

**Keywords:** Alzheimer disease, dementia, metallomics, trace elements

## Abstract

Changes in trace element concentrations are being wildly considered when it comes to neurodegenerative disorders, such as Alzheimer’s disease and Parkinson’s disease. This study aims to present the role that trace elements play in the central nervous system. Moreover, we reviewed the mechanisms involved in their neurotoxicity. Low zinc concentrations, as well as high levels of copper, manganese, and iron, activate the signalling pathways of the inflammatory, oxidative and nitrosative stress response. Neurodegeneration occurs due to the association between metals and proteins, which is then followed by aggregate formation, mitochondrial disorder, and, ultimately, cell death. In Alzheimer’s disease, low Zn levels suppress the neurotoxicity induced by β-amyloid through the selective precipitation of aggregation intermediates. High concentrations of copper, iron and manganese cause the aggregation of intracellular α-synuclein, which results in synaptic dysfunction and axonal transport disruption. Parkinson’s disease is caused by the accumulation of Fe in the midbrain dopaminergic nucleus, and the pathogenesis of multiple sclerosis derives from Zn deficiency, leading to an imbalance between T cell functions. Aluminium disturbs the homeostasis of other metals through a rise in the production of oxygen reactive forms, which then leads to cellular death. Selenium, in association with iron, plays a distinct role in the process of ferroptosis. Outlining the influence that metals have on oxidoreduction processes is crucial to recognising the pathophysiology of neurodegenerative diseases and may provide possible new methods for both their avoidance and therapy.

## 1. Introduction

Dementia is characterized by cognitive deterioration, which is substantial and impacts autonomous, everyday functioning. It is described as a set of symptoms instead of one particular disorder. Neurodegenerative dementias are most prevalent in the older population, while traumatic brain damage and tumours are more frequent in the younger population [1]. Alzheimer’s disease (AD) is a neuron degenerative illness with progression that causes permanent damage to thinking processes, memory, and linguistic abilities. AD is the most prevalent dementia type among elderly patients, with slow progression from mild forgetfulness to the need for total care. The earliest phase of AD is described as amyloid β-protein (Aβ) aggregation in senile plaques, the occurrence of neurofibrillary tangles (NFTs) that are comprised of an unusually phosphorylated tau protein, and a decline in the neuronal number, which all lead to cognitive decline [2]. The only conclusive way to diagnose AD is to perform a brain autopsy of the patient’s brain tissue and ascertain whether the subject had AD or any other form of dementia.

The risk of Alzheimer’s disease depends 60–80% on genetic factors. Scientists have identified more than 40 AD-associated genetic risk loci, and apolipoprotein E (APOE) alleles, such as the ε-4 allele, are said to have the strongest correlation with the disease, especially its common late-onset variant. *APOE* gene mutations are prevalent in 16% of the population. The amyloid precursor protein (APP) genes presenilin 1 (PSEN1) and presenilin 2 (PSEN2), and their mutations, are responsible for early-onset autosomal dominant AD [3].

**Figure 1:**
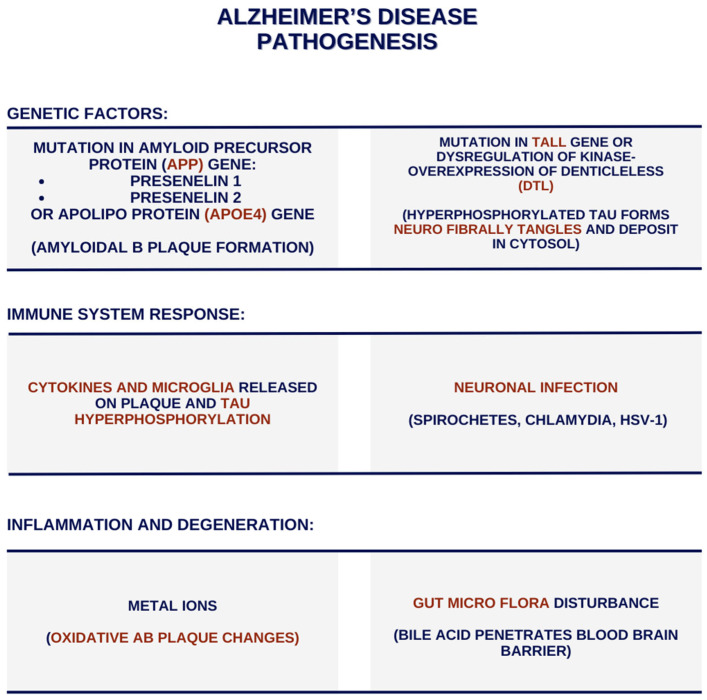
The possible pathogenetic routes of Alzheimer’s disease.

**Figure 2:**
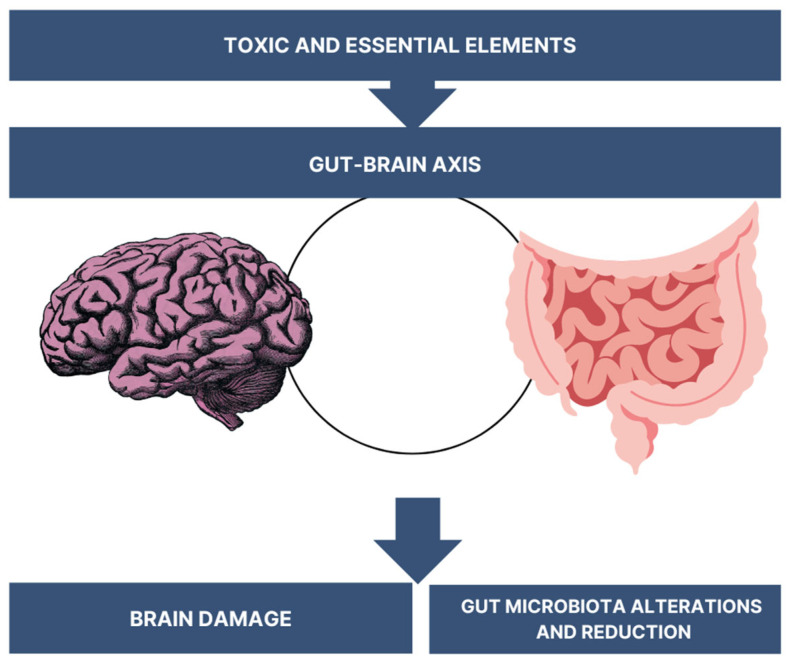
The gut–brain axis that is associated with brain damage.

According to the Aβ plaque theory, aggregations are created and later deposited in various areas of the brain. They are recognised as foreign substances and initiate an anti-inflammatory immune response through microglia activation and cytokine release, leading to cell demise and neurodegeneration. The neurofibrillary degeneration theory suggests that AD is caused by hyperphosphorylation, with the subsided tau proteins having an affinity for microtubules. The hyperphosphorylation of tau causes NFT formation, their deposition in the cell’s cytosol, which is unable to perform its cell-structure-maintaining role, and results in the microtubular loss of kinship [4]. This leads to microtubular instability and modifies axonal integrity. We have identified many trails and areas affected by this disease, such as vascular aberrations, neuroinflammation, and intracellular changes with mitochondrial deterioration and oxidative stress. These processes (presented in the Figure 1) are commonly considered to be the causatives of the disease’s initiation and progression. The correlation between gut microbiota changes and the damage of the brain is presented in Figure 2.

An increased risk of AD occurrence is connected with environmental and metabolic factors, such as a poor diet, reduced physical activity, stress, cerebrovascular diseases and exposure to toxic environmental elements [5]. The oxidative stress hypothesis concludes that the central nervous system is very susceptible to oxidative stress, which can later initiate and promote neurodegeneration [6]. 

Changes in the levels of serum trace elements are common in patients who suffer from psychiatric conditions such as schizophrenia and autism, and are associated with their cognitive decline [7,8].

Substantial studies have shown that we can distinguish trace elements with a positive impact on AD prognosis and progression, like zinc, selenium, intracellular calcium, chromium, and rubidium. In contrast, metals such as iron, copper, lead, mercury, nickel, arsenic and bromine have the ability to not only catalyse oxidation reactions, but also directly impact *APP* gene transcription, initiating Aβ aggregation. Excessive levels of the aforementioned elements might also affect the brain’s ability to utilize Aβ, through the decreased expression of AD-protective lipoprotein receptor-related protein 1 (LRP1) [9]. The described findings serve as a promising starting point for novel AD treatment strategies, and thus should be further understood.

## 2. Trace Elements in Alzheimer’s Disease

### 2.1. Iron (Fe)

The intestine is an area in which, through the action of ferrireductase on the luminal side and the presence of metal transporter 1 on the apical membrane, iron is absorbed [10]. Fe is predominantly located in the regions of the brain that are involved in the supervision of motor neuron activity, exhibiting there up to three times higher concentrations when compared to different areas [11]. The structures that build grey matter are said to have greater iron concentrations when compared to those involved in the formation of white matter [12]. The regions with the highest iron concentrations are the globus pallidus, substantia nigra and caudate nucleus. This heightened iron presence suggests the susceptibility of these structures to the disruptions caused by movement disorders due to fluctuations in iron levels [13,14,15]. As a crucial factor in myelin and neurotransmitter synthesis and a participant in oxidative metabolism, iron possesses a vital role in maintaining optimal brain function. Its functions encompass oxygen transport, glucose metabolism, electron transport, myelin and neurotransmitter synthesis, and DNA replication [11].

As individuals age, there is a rise in the overall iron concentration within the specific brain regions susceptible to degenerative conditions like AD, PD, and Huntington’s disease [16]. The failure to effectively regulate iron homeostasis results in an excess of reactive iron, infiltrating cells like astrocytes, microglia, and neurons. Nonetheless, in over 95% of cases involving AD and PD, the accumulation of iron is comparatively modest, and the neurotoxic mechanism likely entails a synergy between iron and other toxins [17]. Studies consistently provide evidence that elevated levels of brain iron in adulthood adversely impact cognitive abilities. However, higher levels of Fe in the caudate nuclei, hippocampus, and thalamus present a consistent correlation with diminished memory performance. On the other hand, a more coherent association was observed between elevated iron levels in the putamen and diminished general cognitive function [18,19]. Neurodegeneration with brain iron accumulation (NBIA) is defined as a diverse range of conditions that might occur in both children and adults, and is described according to the symptomatic and genetic heterogeneity [20]. The accumulation of iron can either induce atrophy or occur concomitantly with atrophy, subsequently leading to localized damage that may disrupt the brain’s circuitry [21].

Contrary to assessments collected postmortem, evaluations performed in vivo by magnetic resonance imaging (MRI) of Fe levels in brain tissue have revealed elevated iron levels in patients diagnosed with AD [22].

Furthermore, another study showed that patients with AD present with decreased iron levels in the blood and increased ferritin in the cerebrospinal fluid (CSF) compared with healthy controls; baseline CSF ferritin was also reportedly a strong predictor for cognitive deterioration over 7 years in the AD Neuroimaging Initiative cohort [23].

### 2.2. Copper (Cu)

Copper is an essential element and is required as a substrate for various physiological processes occurring in human body cells. Yet, the excessive accumulation of Cu in cells might cause oxidative stress and perturb their proper functioning. Recently, a new form of apoptosis that depends on copper was distinguished: cuproptosis. Copper binds to enzymes that have been lipoylated in the tricarboxylic acid cycle, which later leads to the aggregation of proteins, proteotoxic reactions, and the final demise of the cell [24]. The liver predominantly stores copper, and its excess is eliminated from the body in the form of faeces and through the excretion of bile as unabsorbed metal ions [25]. Intracellular Cu levels are regulated by a complex network of Cu-dependent proteins, including enzymes, and membrane transporters [26].

Wilson’s disease (WD) is a known example of a condition caused by copper overload. WD is an autosomal recessive disorder in which several mutations in the copper-transporting P-type ATPase gene are involved [27]. In this disease, Cu toxicity is believed to be the primary cause of organ damage. Both neurological and neuropsychiatric signs are presented by almost half of patients with the disease, and reportedly nearly all their brain areas test positive for a high copper concentration [28].

AD pathogenesis is commonly associated with changes in Cu serum and cell homeostasis. Cu is believed to interact with key pathogenic factors such as Aβ and tau. Serum-free Cu levels are reportedly elevated in older people, and both total and free Cu levels are increased in the serum of patients with AD [29,30]. A study on senile plaques in patients with AD revealed higher levels of Cu. Moreover, excessive amounts of this metal can directly bind with Aβ peptides, increasing the aggregation of Aβ and neurotoxicity [31]. The sequestration of Cu from Aβ peptides performed in in vitro conditions has a preventive role in their accumulation, and eventually reduces cell death [32]. Furthermore, another essential and notable role of Cu in the process linked to neurodegeneration is microglia activation. In studies performed on microglial cell lines in rodents, copper was able to activate the nuclear factor kappa-light-chain-enhancer of activated B cells (NF-κB) signalling pathway and the release of inflammatory factors, like cytokine tumour necrosis factor-α (TNF-α) and nitric oxide (NO). Additionally, the brain’s physiological ability to utilise Aβ peptides might be reduced by excessive levels of Cu. The expression of LRP1 is reduced by the complexes that copper forms with Aβ, which also decrease the clearance of neurotoxic Aβ and provoke the formation of brain deposits [33].

Many studies have led to the assumption that elevated copper levels in the brain matter might not only contradict the pathophysiological role of amyloid, but also have therapeutic use in disease treatment. There is evidence that copper concentrates with other metals in amyloid plaques. It is reportedly deficient in brain tissue cells, but considerably increased in extracellular plaques. Furthermore, intracellular copper deficiency is said to promote Aβ production, while extracellular Cu2+ abundance can, in acidic conditions, promote Aβ precipitation, oxidative cross-linking and modification. Therefore, the theoretical therapeutic would be able to mobilize extracellular Cu2+ and take it back into the cell [21].

### 2.3. Zinc (Zn)

Zinc is an essential dietary micronutrient that modulates not only the stress response, but also the neurons in excitatory synapses. It influences neurotransmission and the signals obtained from sensual processing. Zinc activates both pro-survival and apoptotic indicating pathways in neurons [34]. Zn^2+^ ions are abundantly distributed in the central nervous system and accumulate in presynapses. Synaptic Zn^2+^ is necessary for processes like neuronal generation, perception, and memory creation.

Disruptions to Zn^2+^ homeostasis might be associated with several disorders affecting the central nervous system, like Alzheimer’s disease, Parkinson’s disease, and depression [35]. Some Zn^2+^-containing transcription factors such as NF-κB and the tumour protein P53 (p53) participate in amyloid precursor protein (APP) synthesis. Zn^2+^ can interact with Aβ and regulate its polymerization [36]. Low concentrations of Zn^2+^ play a protective role and oppose the neurotoxic effect of β-amyloid. On the other hand, increased amounts of ion binding cause fibrillar Aβ aggregation to increase [37]. A growing body of evidence also suggests that elevated levels of Zn^2+^, in cases of advanced AD, may lead to an overly phosphorylated tau protein [38,39]. In another study, Zn abundance visibly reduced motor function, as well as the levels of tyrosine hydroxylase (TH)-positive neuronal cells and the tyrosine hydroxylase protein [40].

Scientists have provided evidence that regular zinc intake is associated with not only a lower risk of cognitive decline, but also a slower deterioration process in patients with mild cognitive impairment and diagnosed AD. The APP/PS1 mouse model of AD was used, with a zinc intake of 35 mg/kg in the control group and 3 mg/kg in the supplement-deficient group. The results showed that without the modification of the amyloid β plaque burden, low levels of zinc enhanced Alzheimer’s-like deficits in male mice. What is more, zinc supplementation inhibited the activation of one of the most important regulators of inflammation—the NOD-, LRR- and pyrin domain-containing protein 3 (NLRP3) inflammasome complex [41].

### 2.4. Selenium (Se)

Selenium is essential for the human organism and plays a significant role in a variety of physiological responses. Fruits and vegetables contain trace amounts of the element; however, its food content is dependent upon regional soil concentrations. Low levels of selenium and selenoprotein have been associated with the decline in cognition and neurodegeneration observed in AD, PD, and HD [42]. Said proteins are well recognized for their anti-oxidative actions. Researchers have reported the beneficiary role of sodium supplementation in neuroinflammation, caused by the intracerebroventricular injection of lipopolysaccharide (LPS). The evidence shows reduced oxidative stress, the downregulation of pro-inflammatory cytokine expression, the improvement of blood–brain barrier integrity, and suppressed glial activation in the mouse brain when supplied with Se (Se-sup). Moreover, Se intake reportedly reduced the apoptosis of neural cells and improved the cognitive processes in mice after the injection of LPS and the supplementation of Se. Said element neuroprotection is likely linked to Glutathione peroxidase 4 (GPX4) and Selenoprotein P (SELENOP) overexpression. Additionally, dietary Se intake is highly related to the SELENOP expression in the mediodorsal thalamic nucleus, hippocampus and lateral habenular nucleus [43].

Furthermore, selenium, in association with iron, plays a decisive role in ferroptosis. Apoptotic promotion by iron occurs thanks to its contribution to Fenton-type reactions and lipid auto oxidation. Selenium, through glutathione peroxidase 4 (GPX4), suppresses the peroxidative processes of phospholipids, and is associated with cellular death. Recent studies have shown another pathomechanism of ferroptosis, which involves ubiquinone outside the mitochondria, NAD(P)H and ferroptosis suppressor protein 1. Unlike GPX4, this system uses peroxyl radicals in membranes, and thus restrains lipid peroxidation [44].

Studies have found decreased selenium levels in patients with AD [45]. Vitamin E, together with seleno-l-methionine, protects against oxidative stress and the toxic influence of β-amyloid [46]. Sodium selenite inhibits amyloid production by decreasing gamma secretase activity [47]. Moreover, as an agonist for protein phosphatase 2A (PP2A), it seemingly reduces the formation of neurofibrillary tangles [48].

In addition, a significant decrease in the possibility of AD (after the inclusion of potential risk factors) has been observed in correlation with selenium levels. An increase in blood selenium was correlated with an almost 1.4 times lower risk of AD [49].

### 2.5. Aluminium (Al)

Aluminium is the most common metallic element naturally found in our environment and is widely used for consumer products and in industrial processes. It is known to have an extensive range of forms in which it can be absorbed, such as food, cookware, as a pharmacological agent, and as an adjuvant in vaccines [50,51]. Although Al is absorbed through the digestive and respiratory system, its intake is restricted by the physiological pH, at which most Al compounds are relatively insoluble, resulting in absorption limitations. This element’s toxic effect is caused by either excessive exposure or direct intake, which might occur via hyper alimentation, dialyses, or the implantation of foreign materials [52].

Aluminium disrupts the homeostasis of other metal elements and increases the production of oxygen reactive forms, leading to cellular death. Al presence and oxidative stress have been reported in microglia and astrocytes [53]. This element affects the cellular redox state by suppressing the activity of the enzymes involved in antioxidant defence, for example, by lowering the activity of super oxide dismutase (SOD) in the cerebrum, cerebral hemisphere, and brain stem [54,55].

Aluminium is known for its neurotoxicity, but the exact mechanism remains unknown [56]. The brain’s aluminium absorption happens due to its diffusivity through the blood–brain barrier and carrier receptors, especially the intracellular trafficking pathway of transferrin [57]. Al exposure is associated with the development of neurodegenerative processes in diseases such as AD, dementia, Amyotrophic Lateral Sclerosis (ALS) and parkinsonism [56]. It accumulates in the hippocampus, frontal cortex, corpus striatum, cerebellum, or brain stem [57]. Aluminium chlorides affect cholinergic and noradrenergic neurotransmission, which mainly manifest as memory and cognitive disfunctions [58]. Furthermore, Al has various adverse effects on the central nervous system, such as impaired axonal transport, the diminished synthesis of neurotransmitters, and the degradation of protein phosphorylation and dephosphorylation [59]. It is said that aluminium influences the inflammatory response in the brain through the proinflammatory cytokines, such as through the elevation of Interleukin 1-alpha (Il-1α) and TNF-α levels [60]. Studies have also reported that Al leads to a decrease in brain-derived neurotrophic factor (BDNF) and neuron growth factor (NGF) expression [61]. Aluminium is able to affect the structure of cytoskeletal proteins like neurofilaments and tau, turning them into aggregates of phosphorylated proteins, as found in AD [62]. Molecular and epidemiological AD evidence has shown the aberrantly localized deposition of aluminium in the brain [56]. To conclude, excessive amounts of Al manifest through neurotoxicity, neurodegeneration, as well as learning and memory function loss [63]. The mechanism behind the accumulation of tau filaments is unclear and its discovery might play a key role in finding new therapeutic strategies [64].

### 2.6. Cadmium (Cd)

Cadmium (Cd), a heavy metal with very high toxicity that accumulates in the body, is one of the most important environmental pollutants. Absorption may happen through the use of tobacco or the ingestion of Cd-contaminated substances. Cd not only has a long biological half-life, but also low excretion from the body, and thus it is predominantly stored in the soft tissues of the human body. As the main storage units of Cd are the liver and kidney, the urinary levels of said trace element remain one of the biomonitoring markers best able to assess the long-term exposure [65]. The neurotoxic effect of Cd has been linked to illnesses with a neurodegeneration component such as PD and AD. Cd affects cell proliferation, differentiation, and apoptosis. However, its impact on cellular toxicity is mostly associated with the Cd-dependent disruption of DNA repair mechanisms and the generation of reactive oxygen species [66]. 

Researchers have studied animal models to assess the effect of the chronic administration of Cd on oxidative stress reactions and the association with AD pathology. Divided into two groups, mice were administered saline or Cd for a duration of three months. Behavioural, molecular, biochemical, and morphological studies and analyses were conducted. Synaptic deficits, along with the intensification of memory loss, were reported in mice chronically injected with Cadmium. Chronic and subacute Cd intake increased reactive oxygen spieces (ROS), suppressed antioxidant enzyme activity, and evoked the stress kinase signalling pathways. These findings suggest that targeting Cd and its influence on oxidative stress, together with amyloid beta pathologies, might form a potential therapy route in AD [67]. 

Cd exposure reportedly greatly impacts the gut–liver axis in the mouse strain most susceptible to neurological damage (with ApoE4), which is supported by an increase in microbial AD biomarkers, reduced blood and gut energy supply pathways, increased hepatic inflammation and the biotransformation of xenobiotics [68].

Another study focused on the connection between AD mortality and blood cadmium levels in a group of elderly people. Scientists used the prospective data from a study conducted between 1999 and 2004, namely the Third National Health and Nutrition Examination Survey and the Linked Mortality File. Around four thousand participants older than 60 years with available blood cadmium data were included in this study. As a result, a comparison showed that participants with high blood cadmium levels, over 0.6 μg/L, exhibited an almost four times higher risk of mortality connected to AD. This shows that environmental cadmium exposure and intake might be a crucial risk factor when it comes to AD [69].

### 2.7. Calcium (Ca)

Calcium and especially its ions (Ca^2+^) are substantial in many physiological and biochemical processes, such as the transduction of signals, neurotransmitter release from neurons, and muscle contraction. Extracellular particles maintain the difference in potential across cell membranes. Excessive Aβ formations are commonly believed to incite the pathogenic cascade of cholinergic neuron degeneration. On the other hand, calcium located in the cells regulates the functions of the neurons, such as their growth and differentiation, as well as the plasticity of the synapses. Hypothetically, activation of the amyloid-generating pathway impacts Ca^2+^ homeostasis and memory-creating mechanisms. The disturbance of Ca^2+^ signalling is caused by the extracellular influx of Ca^2+^ ions and the activation of its release from the intracellular stores [70].

The activity of the neuronal network and its functional connectivity (FC) may be impaired early on in AD pathogenesis. The exact mechanisms still remain unclear. Local neuronal activity in the healthy brain is regulated by astrocytes. Increased FC in the cingulate cortex often exceeds amyloid deposition by several years. Researchers have confirmed the same early cingulate disruption in mice models of the disease. Damage to the network is connected to the decrease in astrocyte Ca signalling. Neuronal hyperactivity and functional connectivity are normalized by the recovery of calcium activity in the astrocytes [71].

Moreover, all the stages of AD are characterized by the dysregulation of calcium homeostasis. The process is connected to mitochondrial failure, oxidative stress, and chronic neuroinflammation. Calcium channels and receptors are reportedly involved in pathophysiological mechanisms at the levels including neuronal and glial cells. The relationship between amyloidosis and the activity of the glutamatergic NMDA receptor (NMDAR) has been extensively studied. Furthermore, L-type voltage-dependent calcium channels, receptor potential channels that are transient, and ryanodine receptors are being considered [72]. Ca^2+^ neuronal homeostasis dysregulation is not only related to age, but also significant in AD pathogenesis, through deficits in synapse induction and the promotion of Aβ plaques, as well as the accumulation of neurofibrillary tangles. The use of molecules specific to Ca^2+^ channels and the insertion of proteins into the plasma and intracellular membranes might improve the disruption of Ca^2+^ homeostasis and become a new method of AD treatment [73].

### 2.8. Lead (Pb)

Recently, significant attention has been directed toward specific multivalent cations as potential neurotoxic agents, in the context of neurodegenerative diseases [74]. Accumulated exposure to lead has been linked to ongoing gradual cognitive function loss, persisting well after a decrease in lead levels in both the brain and blood [75]. It is also noteworthy that urine can be used to replace blood for the assessment of the occupational exposure to lead [76]. Historical exposure to lead is correlated with brain lesions (both global and region-specific ones). The accumulation of lead dosage is linked to an elevated prevalence and severity of white matter lesions, an overall brain volume reduction, as well as diminished global and region- or structure-specific volumes [77,78].

Lead exposure has the potential to induce damage to the white matter of the brain, apoptosis of the cells, as well as alterations in their structure, with synaptic density or connection changes. Some of the observed alterations in cognitive function, typically associated with age-related changes, are likely linked to previous exposure to lead [79,80,81,82,83,84].

Haraguchi et al. investigated the lead levels in both brain tissue that was freshly frozen and in brain tissue fixed with 10% formalin from individuals with diffuse neurofibrillary tangles with calcification (DNTC), using spectrometry with flameless atomic absorption. These findings revealed a significant concentration of the studied metal in these tissues, suggesting that DNTC pathogenesis may rely on lead [85].

Oxidative and endoplasmic reticulum stress, and mitochondrial and ultimately neuronal damage, are caused by lead exposure. Neuronal cells suffer from excitotoxic damage associated with lead exposure and reactive calcium overactivation. Moreover, lead disrupts the homeostasis of essential metals and changes proper metal signalling. These actions ultimately lead to neuroinflammation. Related damage arises in oligodendrocytes, glial and astral cells, and the cerebrovascular endothelium. Extended exposure also leads to changes in epigenetic and epigenetic regulators in the brain matter. It is noteworthy that early-life lead exposure might have latent effects [86,87].

One study showed that young rats exposed early to lead had an increased expression of APP and β-secretase 1 (BACE1), which further led to amyloid accumulation and the formation of plaques in the brain cortex and hippocampus. When combined with arsenic and cadmium exposure, lead further not only enhanced the expression of said genes, but also amyloid β production [88].

The disruption of LRP-1-mediated clearance in acute lead exposure causes an increase in brain tissue and cerebrospinal fluid Aβ accumulation [89].

### 2.9. Magnesium (Mg)

Magnesium (Mg)’s role in the nervous system is signal transmission, neuromuscular conduction, and the preservation of blood–brain barrier integrity. Mg affects many biochemical mechanisms that are fundamental for neuronal function and synaptic plasticity stability. It controls the viscosity of the cell membrane and is an antagonist of calcium. Moreover, it prevents the excessive neuronal excitation, called excitotoxicity, that can result in cell death [90]. Scientists have studied the involvement of Mg in the pathogenesis of epilepsy, migraine, AD, PD, stroke, anxiety, and depression. The contribution of Mg to the mechanisms of neurological diseases implies that the metal might be taken into consideration as a potential remedy for many neurological conditions [91].

Mg deficiency contributes to systemic low-grade inflammation, and neuroinflammation is an indication of neurodegenerative disorders. The collected data have presented evidence that links the imbalance of magnesium levels with multiple sclerosis, AD and PD [92].

A decrease in Mg levels has been reported in various tissues of patients with AD, and has a negative correlation with the deterioration of their clinical state. Said element was also found to modulate the molecular processes involving the amyloid-β precursor protein. However, although patients suffering from AD have lower magnesium concentrations, their deficiency levels cannot be accurately evaluated. Slutsky et al. reported that after long-term supplementation, the Mg^2+^ concentration in cerebrospinal fluid increased only by 15% [93]. The blood–brain barrier keeps at bay the daily fluctuations in blood magnesium and their possible impact on the brain tissue. The hippocampus and its synapses are highly influenced by the smallest changes in the non-cellular Mg^2+^ concentration. When compared with other Mg^2+^ compounds, magnesium L-threonate supplementation could significantly increase Mg^2+^ levels in the cerebral matter. In summary, recently conducted research suggests that magnesium supplementation or restoration may be a novel approach to AD treatment [94,95].

### 2.10. Mercury (Hg)

Mercury (Hg), a toxin that can commonly be found in the environment, appears there mainly thanks to coal burning. The eruption of volcanoes causes copper to be released in the form of a natural vapor or rock. Humans are exposed to its most toxic form, methylmercury, by the ingestion of fish. Methylmercury’s toxic effects show predominance in the brain. Even trace amounts of mercury are said to elicit harm with long-term exposure. Mercury reportedly affects the hallmarks of AD, such as the formation of senile plaques outside the cells and neurofibrillary tangle synthesis within them [96].

Dysfunctional signalling in both AD and exposition to Hg seem to be connected with the appearance of the same substances. Those factors include arachidonic acid, homocysteine, dehydroepiandrosterone sulphate, melatonin, glutathione, acetyl-L carnitine, hydrogen peroxide, glucosamine glycans and high-density lipoprotein [97]. AD-affected brain structures have reportedly demonstrated elevated Hg levels in much of the performed research. Autopsied AD patients studied by Ehmann et al. presented higher levels of Hg in the brain matter, when compared to healthy control subjects [98]. Moreover, twice as much Hg content in the brain tissue of AD patients was reported by yet another study, and an increase in the metal’s concentrations was observed in various compartments of the cell, such as the mitochondria and nucleus.

In the investigation, the CSF of AD patients showed a significant elevation of Hg levels. It is important to mention that whether this increase was associated with AD or age is questionable [99].

There is also a contradiction when it comes to data regarding blood Hg levels in AD patients. The results of research performed by scientists like Basun et al. and Hock et al. have shown an increase in both plasma and blood Hg levels in patients suffering from AD, in opposition to age-matched healthy controls [100]. Conversely, a two-time decrease in the blood serum copper content was demonstrated by Paglia et al. [101]. Nails and hair analysis showed reduced levels of copper in AD patients in comparison to the control group.

Recent studies have highlighted the relationship between Hg and AD. The interaction ranges from the degeneration of the neurons, cell death, the malfunction of mitochondria, the disturbance of the gastrointestinal microflora, to gene expression alterations [102].

### 2.11. Arsenic (As)

Arsenic (As) is a naturally occurring environmental risk factor, with a high representation in drinking water. As is believed to be toxic to neurons and to impair cognitive and memorial functions. Exposure to As and its metabolites leads to ROS formation, inflammation, the dysfunction of the mitochondria, cell death, and disturbances in not only protein homeostasis, but also in calcium signalling. Exposure to arsenic causes the over phosphorylation of the tau protein and APP activation through transcription [103].

The neurotoxicity of As and Hg stimulates free oxygen species formation and a decrease in antioxidant defences. Additionally, mercury has the ability to change the property and structure of β-amyloid when arsenic contributes to its accumulation. Moreover, astrocytes intoxicated with mercury and arsenic initiate neuroinflammation. The symptoms observed during intoxication with mercury and arsenic have many similarities to those observed in AD and PD, and their pathogenetic mechanisms, such as oxidative stress and neuroinflammation, coincide [104].

Studies have shown that a prolonged and regular exposure to arsenic is able to change numerous arsenic-regulated brain regions (related to cognitive impairments) in rodents. The BBB clearance route is also affected by As. Arsenic increases the expression of APP and the processes led by presenilin and β-secretase. What is more, this trace element has an effect on DNA repair routes [105]. 

Studies have reported that exposure to Arsenic (AS) leads to apoptosis mediated by nitric oxide (NO). Arsenic treatment is followed by S-nitrosylation (SNO) reprogramming in the cortex. A study of mice with different drinking water supplementations of Sodium Arsenite (SA) was conducted. Bioinformatics analysis showed the significant S-nitrosylation enrichment of mitochondrial respiratory processes and antioxidant defences, the regulation of transcription, the maintenance of the cytoskeleton, and the regulation of programmed cell death. Dysfunctions similar to those observed in autism spectrum disorder (ASD) and AD were observed through behavioural studies, and the genetic mutations causing ASD and AD were reported by the molecular convergence of the neuronal toxicity caused by SA [106]. 

In the conducted studies, AD patients presented with a greater urinary arsenic content when compared to controls. Arsenic and its metabolite contents in urine showed a possible association with an increased risk of AD. However, the correlation between cognitive function decline and arsenic levels is not wholly consistent. Serum arsenic analyses did not report a correlation with AD, while a greater amount of said element was found in the nails and hair of AD patients [49].

### 2.12. Bromine (Br)

Bromine is an element that occurs naturally and is widespread in the form of flame retardants, pesticides, and water treatments. Scientists investigated the association of brain bromine concentrations with AD neuropathology. The study was conducted in 215 deceased participants and, through the analysis of neuronal activation, the bromine levels in brain tissue were measured. The assessment included a search for diffuse and neuritic plaques, as well as neurofibrillary tangles, in various brain regions. The linear models with function regression measurement showed that higher bromine levels in brain tissue were related to more advanced AD pathological processes [107].

The decabromodiphenyl ethane (DBDPE), an alternative to brominated flame retardants, is widely used and therefore has been oftentimes present in the environment. It has been demonstrated that said chemical causes a decline in locomotor abilities and, thanks to a reduction in transthyretin, Aβ binding worsens amyloid deposition [108]. 

Moreover, it has been reported that exposure to 1-bromopropane (1-BP) induces the depletion of cognitive abilities. Evidence suggests that both N-methyl-D-aspartate receptors (NMDARs) and neuronal inflammation play a significant role in learning and memory deficits, as observed in diseases characterized by the degeneration of neurons. Male rats were administered with the noncompetitive NMDAR antagonist MK801 before being intoxicated with 1-BP. An evaluation of their cognition was performed, and their brains were dissected and analysed immunologically, chemically, and neuropathologically. Spatial learning and memory were reportedly impaired in the intoxicated group, and structural transformations showed neurodegeneration in the hippocampus and cortex. Furthermore, MK801 improved both the cognition impairments and brain matter changes induced by 1-BP. Consequently, MK801 inhibited microglial activation and release of pro-inflammatory cytokines. Said findings might provide a potential treatment when it comes to 1-BP poisoning [109,110].

### 2.13. Chromium (Cr)

The dietary intake of this trace element is derived from food processing with stainless steel equipment. Chromium is reportedly involved in regulating carbohydrate and lipid metabolism through an increase in insulin’s efficacy. In the bloodstream, it travels bound to the transferrin and is delivered to targeted cells through the process of endocytosis. Chromium (6+) and its compounds have high carcinogenic and mutagenic properties, especially when inhaled and ingested in large amounts [111].

In one study, chromium picolinate (CrPic) was tested at various doses, with the use of rivastigmine as a control drug. Streptozocin caused congnitive dysfunction, which was later evaluated with the use of behavioural tests. Treatment with CrPic caused a reduction in the cognitive deficit and was confirmed through tests of behaviour, antioxidant enzyme estimation, oxidative and nitrosative stress, as well as the cholinergic and activity of mitochondria. Streptozocin-induced neuroinflammation was made apparent by increased TNF-α, IL-6, and CRP levels, and ceased with the supplementation of CrPic. Moreover, insulin signalling was reportedly improved by CrPic treatment, and was revealed by the increased expression of associated genes such as *IRS-1* and *PI3-K.* In conclusion, CrPic has the potential to reverse AD-related pathological processes, with the improvement of memory, a reduction in oxidative stress and neuroinflammation, and insulin signalling upregulation. Another study reported that higher chromium, together with a lower selenium blood content, is associated with a significant rise in the risk of AD when compared to patients with adverse levels of the mentioned elements [49,112].

### 2.14. Manganese (Mn)

Manganese is a significant trace element for body homeostasis. Its deficiency occurs rarely because it has sufficient levels in food [51,52]. Moreover, manganese can be inhaled as air particles and absorbed through the skin. Fuel additives or fungicides can also contain Mn, and can be a source of manganese entering the body via the respiratory system. Excess amounts of Mn from contamination or occupational exposure present toxic activity [51]. When orally administered, Mn transforms to Mn^2+^ in the presence of gastric acid, then is oxidized to Mn^3+^ and transported bonded to tissue factor (TF) through the blood–brain barrier. Zinc transporters are also known to take part in Mn’s absorption control [113]. In the human body, manganese supports neurotransmission, cellular metabolism, antioxidant defence and take parts in inflammatory responses [114,115]. It is also a cofactor of several enzymes. There are a few Mn-dependent enzymes, like arginase, agmatinase and glutamine synthetase, that take part in neurotransmitter synthesis [116].

Because of its similar chemical structure and activity to Fe, Mn influences the iron-responsive element-binding protein (IRE-IRP) pathways, leading to expression changes in Fe-related proteins. A high Fe intake decreases blood Mn levels and anaemia increases intestinal Mn absorption. A diet rich in Mn leads to lower Fe plasma levels, but elevated transferrin levels and total iron-binding capacity (TIBC) [117].

Due to its role, both a deficiency and excess amount of Mn can be harmful, causing cognitive dysfunction or “manganese” neurotoxicity connected to basal ganglia system failure. It then results in severe neurological illness, with extrapyramidal symptoms like hypokinesia, rigidity, and tremor similar to PD [118]. The overabundance of Mn accumulates in the striatum of the basal ganglia, globus pallidus, cerebellum, and hippocampus [119]. Excessive exposure induces cell enlargement, hepatocerebral disorders and a physical alteration, which may also be seen in AD.

Manganese poisoning deteriorates the activity of basic glial cells and the metabolic pathways of the astrocytes, causing impairment that can be either direct or indirect [120]. Studies have connected increased Mn concentrations with elevated plasma levels of amyloid-like protein-1 and Aβ peptides, the hallmark of AD. Furthermore, research by Chtourou and Fetoui et al. suggests that the activity of the cholinergic enzyme acetylcholinesterase (AChE) may be altered by excessive levels of Mn. Non-human primate PET images show that, in the frontal cortex, Mn causes alterations in the D1- dopamine receptor (D1R), which might cause deficits in memory and attention in subjects exposed to Mn [121].

### 2.15. Nickel (Ni)

Nickel is a metal that naturally appears in the crust of the earth. Ni is widely used in modern industry. Inhalation and ingestion are the main exposition routes. Substantial amounts of nickel may be deposited and accumulated through exposure in the working environment and diet.

The environment is a known determinant of brain health, and it is associated with a higher risk of neurodegeneration. Nickel is one of the environmental metals that negatively impacts human health. Patients affected by neurodegenerative disorders reportedly present elevated Ni levels in the blood, serum, CSF and brain tissue. Such findings are said to be a consequence of contact with contaminated air, food, and water, as well as direct skin exposure. This contact increases lipid peroxidation marker concentrations and causes a decrease in the activity of antioxidative systems. A reduction in exposure to the contaminant may be key to lowering the incidence of neurodegenerative disorders [122].

Alzheimer’s disease’s main pathological components include the tau protein, a microtubule-associated protein whose aggregation is modulated by metals such as zinc, copper, and iron. The performed study showed that nickel and its synthetic morpholine conjugate induce the degradation of tau, and prevent its accumulation, as evidenced by electrophoresis in sodium dodecyl sulfate–polyacrylamide gel (SDS-PAGE) and imaging using a transmission electron microscope (TEM) [123].

A recent study also showed that the aggregation of the amyloid β40 peptide in vitro was enhanced by the addition of zinc and nickel ions in acidic conditions. The results indicate that nickel ions have the ability to enhance Aβ peptide aggregation and that nickel chelation inhibits said process. Hence, dimethylglyoxime (DMG)-mediated Ni-chelation is a possible and promising method of inhibiting or slowing down amyloid mass formation [124,125].

### 2.16. Rubidium (Rb)

Rubidium is an alkali metal, and its compounds have various chemical and electronic applications. It is not a known nutrient, but its ions are known to have similar properties and the same charge as potassium ions [126].

Alzheimer’s disease is characterized by the intracellular potassium loss of compartmentalization. This process indicates membrane damage and mitochondrial dysfunction. Researchers examined the potassium and rubidium in the blood, cerebrospinal fluid, and brain tissue of those with Alzheimer’s disease and a group of healthy control subjects. A notable decrease in both potassium and rubidium levels was observed in all intracellular cerebral compartments. The erythrocytic and CSF rubidium levels did not significantly differ according to the disease state, and rubidium was slightly decreased in AD-affected patients in comparison to the healthy controls. The systemic decrease in cortical potassium and rubidium levels indicate a possible internal energy crisis within the brain [127].

Mercury, iron, rubidium, selenium, and zinc levels were measured in the pituitary gland. The aim of the study was to assess the potential difference in the environmental Hg exposure between AD patients and control subjects, as the pituitary gland is believed to be a good predictor of Hg exposure. No significant differences were noticed with regard to the mentioned elements. The final results suggest that no excessive exposure to Hg was reported in AD patients [128].

### 2.17. Strontium (Sr)

Strontium is an alkaline earth metal that is mainly mined from its mineral forms—celestine and strontianite.

One study reported that elevated Al, Sr, Fe, Ba, Mn cation levels, combined with Mg and Ca deficiency, are present in the diet of Chamorro populations. Said populations are heavily affected by the incidence of illnesses such as AD, PD, and motor neuron diseases.

A fivefold increase in ‘magnetic susceptibility’ readings was demonstrated by the regional soils when compared with nearby regions that were disease free.

After the impairment of the barrier between the gut and brain, the increased uptake of the aforementioned elements into the brain, which lacks magnesium and calcium, leads to metal substitution at their binding domains. This causes a disruption in systems dependent on magnesium and calcium. As a result, the rogue metals lead to sulphate chelation and crystal proliferation, with the ultimate collapse of proteoglycan-mediated cell signalling, which is important for neuronal growth and structural integrity maintenance. The rogue metals overload enables the spread and proliferation of metal–protein conglomerates. To sum up, the emission of the magnetic field might initiate the progressive pathogenesis of neurodegeneration, which is mediated by free radicals [129].

## 3. Alzheimer’s Disease Therapies That Might Target Elements

Metals and the oxidative damage mediated by them are a significant contributor to the ethology and pathogenesis of AD. Iron, aluminium, zinc, and copper brain tissue levels are dysregulated in AD patients. Chelators are compounds that are able to form a connection with metal ions and have a regulative role in their bodily concentrations. The chelator desferrioxamine, used in cases of iron overload, has shown some benefits when used in AD patients, but it has many adverse effects and no tissue-specific targeting. Chelators conjoined with nanoparticles exhibit an ability to penetrate through the barrier between the blood and brain, chelate metals, and exit through the same barrier with the assigned metal ion complex. Said action route may, in conclusion, be a safer and more effective way to reduce the neural metal load. It can further cease the harmful effects of oxidative damage. Moreover, studies of Iodochlorhydroxyquin (clioquinol) show that this copper and zinc-specific chelator can easily penetrate the BBB and dissolve amyloidal plaques. A therapy using clioquinol in AD patients resulted in slower cognitive decline, compared with that in controls. On the other hand, clioquinol has been withdrawn from the antibiotic market due to its association with subacute myelo-optic neuropathy. Hence, clinical investigations of recently developed second-generation clioquinol are important [6,130,131,132]. 

Both AD itself and its prodromal form—Mild Cognitive Impairment (MCI)—are characterized by copper metabolic imbalance and higher-than-normal values of copper not connected to ceruloplasmin. Zinc is involved in intestinal cell metallothionein induction, with intestinal tract copper absorption blocking, and in maintaining the physiological levels of non-ceruloplasmin copper in the body. In patients affected by AD, non-ceruloplasmin copper levels are effectively decreased by zinc, and the metal shows the potential to improve cognitive function. What is more, no considerable side effects have been reported [133]. 

Another therapeutic route involves combined probiotic and selenium supplementation. It is said to correct metabolic abnormalities and cease inflammation and oxidative stress. Researchers found out that after 12 weeks of supplementation with probiotics and selenium, patients with AD saw an improvement in their mini mental state examination overall score [134].

Furthermore, in a different study, scientists took into consideration that patients with AD present zinc deficiency when compared to their age-matched controls. The neuronal protection is deeply dependent on zinc levels. A double-blind trial, with a duration of 6 months, tested a new zinc formulation in AD patients. The results showed that in patients that were 70 years and older, cognitive decline was protected by zinc therapy when compared to controls who were administered the placebo drug [135].

**Figure 3:**
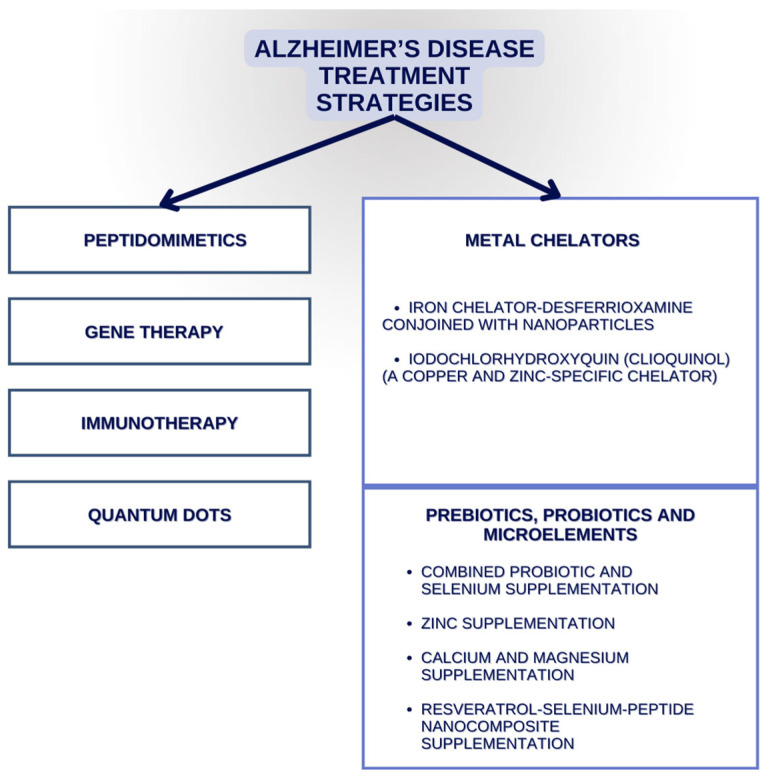
Selected Alzheimer’s disease treatment strategies.

Resveratrol (Res), a grape skin extract, has neuroprotective properties, but because of its low bioavailability in vivo, a Res–selenium–peptide complex was developed. Its main role was to enable the application of Res in aluminium chloride and in a mice model with d-galactose-induced AD. The oral administration of said substance was observed to improve cognition through a decrease in Aβ aggregates and the inhibition of Aβ deposition in brain regions such as hippocampus. Moreover, it is believed to decrease ROS formation and improve the activity of antioxidative enzymes. Additionally, it down-regulated Aβ-induced neuroinflammation and alleviated gut microbiota disorder (particularly targeting bacteria such as *Alistipes*, *Helicobacter*, *Rikenella*, *Desulfovibrio*, and *Faecalibaculum*) [136]. We present a selection of both already known and novel AD treatment strategies in Figure 3.

## 4. Trace Elements’ Abbreviations in Other Dementia Disorders

The term ‘dementia’ encompasses the impairment of the cognitive domains, including language, perception, memory, attention, social cognition, and executive function.

Dementia involves a heterogeneous cluster of disorders including AD, frontotemporal lobe dementia, vascular dementia, and Lewy body dementia, which can be further divided into Lewy bodies dementia (LBD) and Parkinson’s disease dementia (PDD). Frontotemporal dementia (FTD) is often associated with excessive aluminium exposure. Elevated levels of selenium may also be linked to FTD pathogenesis. PDD has been correlated to iron as well as elevated levels of manganese and zinc. The role of copper concentrations in said disease remains unclear and needs further investigation. The correlation between metals and LBD requires more research [137,138].

Amyotrophic lateral sclerosis (ALS), with symptoms similar to AD, has been epidemiologically linked to the occurrence of higher levels of mercury and lead, and to a deficiency of zinc and selenium [139,140].

### 4.1. Lewy Bodies Dementias

The elevation of Fe levels might possibly correlate with an increased incidence of PDD [141,142]. Fe is said to mainly deposit in the substantia nigra and globus pallidus, leading to the degeneration of dopaminergic neurons. This may be further related to increased α-synuclein aggregations and decreased ceruloplasmin, leading to high oxidative stress [143].

A correlation between the deposition of Fe and gene expression in neuronal cells was reported by a different study [144]. The overexpression was then involved in the detoxification of metals and the regulation of the synaptic functions, which supports the theory that a reduction in Fe levels is necessary. On the contrary, an investigation by Ajsuvakova et al. [145] performed on PD patients and healthy controls with samples collected from the subjects’ hair, serum, and urine detected no changes in total Fe concentrations. Willkommen et al. [146] obtained similar results in CSF and total plasma samples. Further research is needed to determine whether there exists a true relationship between Fe and PDD.

A decrease in serum Zn concentrations in PD patients was observed in the study conducted by Kim et al. [147]. Moreover, these findings were positively linked to the subjects’ age. Adversely, a hair study showed elevated Zn levels in older individuals with PD [148]. A later follow-up study reported the co-appearance of psychiatric disorders such as depression and high Zn concentrations [149]. On the contrary, other studies have found no difference in the serum levels of Zn in the urine, hair, CSF, and serum of PD patients compared to healthy controls [145,146].

Mn deficiency presents very similar motor symptomatology to PD. In numerous studies, an increase in Mn concentrations (in the form of albumin complexes) has been associated with a higher PD incidence [150,151]. However, Mn overabundance might be also related to the appearance of motor disorders without the existence of cognitive impairment. The authors state that distinguishing between Mn-induced motor neuron impairment and the motor signs of PD is significant [145].

Oxidative stress mechanisms and ROS formation might be activated by free copper. This metal leads to the degeneration of neuronal cells by binding with alpha–synuclein aggregates [146]. Kim et al. [147] described not only low serum in PD patients, but also their negative relation with MMSE scores, and consequently, with greater cognitive decline.

Lee et al. [152] found that the highest As soil contaminations correspond with the highest prevalence of PD. Thus, As exposure in the working environment is strongly related to the development of PD. Aggregates of α-synuclein can be formed by arsenic in murine models, which can further lead to the creation of Lewy bodies. Mercury is another element with the ability to bind with the mentioned α-synuclein. It is also associated with the formation of Lewy bodies in affected brain regions. A correlation between Hg accumulation and motor symptom intensification in PD patients has been reported by scientists [153]. Moreover, elevated concentrations of Pb have been found in the tibia of PD patients, and mitochondrial DNA biomarkers are used to measure tibial Pb contents [154]. In a study by Dos Santos et al., the control subjects’ hair had seemingly elevated Ca levels when compared to PD patients [148].

### 4.2. Frontotemporal Dementia (FTD)

Frontotemporal dementia involves the progressive degeneration of the brain’s frontal and temporal lobes, appears earlier in the lifetime and can be distinguished into various subtypes, with the most widely known and described being the behavioural variant and the variant affecting language—the aphasic one. We can distinguish Pick bodies in the behavioural variant of FTD (known as Pick’s disease due to these bodies), which are spherical cytoplasmatic inclusions in affected cells. They are mainly built by tau fibrils and other protein products like tubulin and ubiquitin. We can detect a link between tau-positive FTD and Parkinsonism related to chromosome 17 (FTDP-17), which are mutations in the chromosome 17 *MAPT* gene that plays a role in encoding the tau protein [155].

In a study conducted on a group of 45 patients with the behavioural variant of the disease and 35 patients with the aphasic one, the serum Cu and ceruloplasmin concentrations were measured against healthy controls [156]. No significant differences among the Cu metabolism markers were reported.

Another study by Adani et al. [157] found a link between a higher prevalence of FTD and Al exposure in the workplace. Moreover, the prolonged supplementation of Se was also reported as a risk factor in the FTD development process.

### 4.3. Amyotrophic Lateral Sclerosis (ALS)

Amyotrophic lateral sclerosis is a disorder characterized by the loss of motor neurons, which further results in the degeneration of the muscles and ultimately leads to patient death. The major pathology observed in said disease is the accumulation of cytoplasmic and nuclear inclusions of TAR DNA-binding protein 43 (TDP-43) [139].

Said protein occurs in the cellular nucleus and typically binds with DNA and RNA. It contributes to not only gene expression, but also the cycle regulation of mRNA. Hyperphosphorylated and ubiquitinated inclusions of TDP-43 aggregates in nerve cells’ cytoplasm have been detected in most cases of sporadic ALS [140]. The disease pathomechanisms seemingly include an imbalance in metal concentrations and environmental metal exposure.

An increased risk of ALS has been related to not only age, gender, diet, or smoking, but also to excessive contact with heavy metals, such as lead and mercury. On the contrary, scientists have detected lower levels of zinc and selenium in patients with ALS; hence, its possible supplementation may play a significant role in future treatment development [139,140].

## 5. Conclusions

Numerous evidence implies that metal interactions with Aβ might elevate the production of ROS and therefore aggravate oxidative stress reactions. The abundance of Fe^3+^ and Cu^2+^ leads to the generation of H_2_O_2_ and ROS. They further affect the peroxidation of lipids, and the formation of DNA and proteins. The changes in Aβ conformation induced by Zn^2+^ may provide protection by suppressing the oxidation between metals and Aβ. High concentrations of manganese, copper and iron can lead to intracellular α-synuclein aggregation, initiating synaptic and axonal transport disorders. Selenium, when associated with iron, plays a significant role in the apoptotic process related to ferroptosis. What is more, selenium and probiotic supplementation has been linked to the possible prevention of Alzheimer’s disease. The concentrations of all of the aforementioned metals in different bodily tissues and fluids are presented in Table 1.

Chelators, together with nanoparticles, can penetrate the barrier between the blood and brain, chelate metals at the site of their action, and exit through the same barrier with the corresponding particles. Such a process may further reduce metal load and the effects that oxidative damage has on neurons. Resveratrol, a grape skin extract, has neuroprotective properties and when conjoined with a selenium–peptide nanocomposite, decreases Aβ aggregation, inhibits Aβ deposition and increases the activity of antioxidative enzymes.

In conclusion, understanding metals’ influence on oxidoreduction homeostasis may provide possible new targets for the prevention and treatment of neurodegenerative diseases. 

## Figures and Tables

**Table 1:** Concentrations of trace elements in Alzheimer’s disease. Up arrow—increase. Down arrow—decrease.

Concentrations of Trace Elements in Alzheimer’s Disease			
Element	Brain Tissue	Blood	Other (Cerebrospinal Fluid/Urine/Hair/Nails)
**Iron**	↑ In vivo	↓	↑ Ferritin in cerebrospinal fluid
**Copper**	Deficient in the brain tissue cells, increased in the extracellular plaques	↑	n/a
**Zinc**	↓	↓	n/a
**Selenium**	↓	↓	n/a
**Aluminium**	↑	↑	n/a
**Cadmium**	↑	↑	↑ urine
**Calcium**	Ca^2+^ signalling disturbance	n/a	n/a
**Lead**	↑	↑	↑ urine
**Magnesium**	↓	↓	↓ Cerebrospinal fluid
**Mercury**	↑	↑ ↓	↓ Hair and nails
**Arsenic**	↑	n/a	↑ Urine, hair and nails
**Bromine**	↑	n/a	n/a
**Manganese**	↑	↑	n/a
**Nickel**	↑	↑	↑ Cerebrospinal fluid
**Rubidium**	↓	↓	↓ Cerebrospinal fluid
**Strontium**	↑	↑	n/a
**Chromium**	↑	↑	n/a
